# Critical care outcome of pulmonary artery hypertension

**DOI:** 10.1186/cc9914

**Published:** 2011-03-11

**Authors:** A Philips, J Hurdman, B Batuwitage, D Kiely, G Mills

**Affiliations:** 1 Sheffield Teaching Hospitals, Sheffield, UK

## Introduction

Critical care (CC) outcome in pulmonary hypertension (PH) is not well documented, but is generally assumed to be poor. We therefore investigated the critical care outcome in 8 years of noncardiothoracic admissions to a PH supraregional centre.

## Methods

We recorded the following data in PH patients admitted to CC: demography, aetiology, cardiovascular parameters including NYHA classification, R heart catheter and shuttle test distance (most recent assessment) along with organ support data. We recorded the length of stay (hours) in CC, CC and hospital outcome, 1-year survival and eventual outcome.

## Results

Forty-seven patients were admitted (33 women), six required invasive ventilation, another six required non-invasive ventilation (NIV), 18 needed inotropic support and nine required CVVH. For survival to discharge, ROC analysis of shuttle distance demonstrated an asymptotic significance of *P *= 0.04 and an area of 0.71 (95% CI = 0.52 to 0.91) with 83% sensitivity and 65% specificity for a shuttle of 255 metres. Those with a shuttle over 255 metres had an average unit survival of 94%, 88% at hospital discharge and 47% at 1 year. Those below 255 metres had an average survival of 56%, 44% and 33%, respectively. Five out of six invasively ventilated patients died in hospital, but one lived for more than a year after discharge. Three out of six patients receiving NIV died in hospital but three lived for more than a year after discharge. Seventeen out of 18 who required inotropic support were dead at 1 year and 74% died before hospital discharge. For CVVH, five died and four lived. Overall survival: 64% survived to leave CC, 55% were discharged home alive and 34% were alive at 1 year. See Table 1.

**Table 1:** 

	Age	NYHA	**SpO**_**2**_	RA	MPAP	CI	PVR	MVsats	Shuttle	LOS
Average	44	2.9	93	13	50	2.8	751	61	231	126
SD	18	0.6	6	9	14	1.1	324	12	125	154
Median	43	3	93	10	49	2.8	689	65	255	72
25th centile	29	3	92	5	40	2.0	515	54	92	43
75th centile	59	3	96	19	58	3.2	997	68	96	165

## Conclusions

More than one-half of PH patients admitted to CC survive to be discharged home. Shuttle distance gives an indication of likely average survival.

